# Classification of Mycobacterium tuberculosis DR, MDR,XDR Isolates and Identification of Signature MutationPattern of Drug Resistance

**DOI:** 10.6026/97320630015261

**Published:** 2019-04-15

**Authors:** Akshatha Prasanna, Vidya Niranjan

**Affiliations:** 1 Department of Biotechnology, Rashtreeya Vidyalaya College of Engineering,Mysuru road,Bangaluru,India

**Keywords:** *Mycobacterium tuberculosis*, Next Generation Sequencing (NGS), Antimicrobial Resistance (AMR) Prediction

## Abstract

*Mycobacterium tuberculosis* - a global threat, the recent breakout in MDR-TB and XDR-TB has challenged researchers in diagnosis to provide effective treatment. The main objective to combat drug resistance is to provide rapid, reliable and sensitive diagnostic methods in health care centres. This study focuses on development of an effective pipeline to identify drug resistance mutations in whole genome data of Mycobacterium tuberculosis utilizing the Next Generation Sequencing approach and classification of drug resistance strains based on genetic markers obtained from TGS-TB, tbvar and TBDReamDB. 74 isolates are characterized into 20 DR-TB, 16 MDR-TB, 16 XDR-TB and 6 nonresistant strains based on known drug resistance genetic markers. Results provide mutation pattern for each of the classified strains and profiling of drug resistance to the group of anti-TB drugs. The presence of specific mutation causing resistance to a drug will help set the dosage levels which play an important role in the treatment. Findings on amino acid changes and its respective codon positions in candidate genes will provide insights in drug sensitivity and a way for discovery of potent drugs. The implementation of these approaches in clinical setting provides rapid and sensitive diagnostics to combat the emerging drug resistance.

## Background

Tuberculosis (TB) is an infectious disease caused by *Mycobacterium tuberculosis* and has plagued humans since antiquity. The discovery of antibiotics brought a revolution in Tuberculosis Chemotherapy, which started, in 1943with Streptomycin, followed by advent of many potent anti-TB drugs. The implementation of these drugs in tuberculosis therapy immediately resulted in a drastic reduction of TB incidence all over the world. TB was considered to be no longer a public health concern in many developed countries until the outbreaks of multidrug resistant strains in 1980s 1. According to the recent TB report, an estimated 10 million people were infected worldwide in 2017. TB related death was found to be 1.3 million worldwide, making it the largest single infectious cause of death 2. 

*M. tuberculosis* has evolved to emerge as drug resistant strain that has resulted in the restriction of TB chemotherapy which pose an urgent public health problem and requires rapid intervention. The strains were initially resistant to single drugs, have now evolved with sequential accumulation of resistance mutations which has led to the emergence of Multi-Drug Resistance strain (MDR-TB), Extensively Drug Resistance (XDR-TB) and most recently, totally drug resistant (TDR) strains. First-line drugs, which are commonly used for treating tuberculosis such as Rifampicin, Isoniazid, Pyrazinamide and Ethambutol, are becoming ineffective due to mutations in certain genes. These genetic markers are essential for the identification and classification of drug-resistant strains and most importantly give scientists an opportunity to design drugs, which counteract the effects of these mutations. MDR-TB shows resistance to at least one of the two most potent drugs: isoniazid (INH) and rifampicin (RIF). The emergence of XDR-TB resistance is due to having developed resistance to both rifampicin and isoniazid, as well as to fluoroquinolones and at least one of the second-line drugs (i.e., kanamycin, capreomycin, or amikacin) 3. Infections with XDR strains are essentially incurable by the currently available TB drugs. Therefore, these resistant strains of M. tuberculosis pose a serious threat to global control of TB. Alternative treatment strategiesare the need of the hourto tackle the current epidemic of drug resistant TB. Understanding the drug resistance patterns will pave the way to develop new diagnostics and right treatment regime. Genetic markers whose presence confers a high level of probability of drug resistance would be most useful as a diagnostic tool. To identify drug resistance in tuberculosis is to look for catalogue of genes are known to be related with resistance to a particular drug 4. With the motive of identifying drug resistance in a shorter span of time and for rapid screening of multidrug-resistance markers, various molecular approaches have been recommended in the recent times. The current generation NGS analysis helps to identify mutations and is found to be important to understand their effect on drugresistance. The advancement in sequencing technology has provided the whole genome sequencing of Mycobacterium, which gives insight into complete mutation analysis for finding the drugr esistance pattern. Large-scale Whole Genome Sequencing (WGS) is indeed cost effective, thus providing a relatively affordable and faster analysis alternative to analyse drug resistance 5.

## Methodology

### Data retrieval:

The NGS whole genome sequencing paired-end data of Mycobacterium tuberculosis was procured from NCBI-SRA database. The data isfreely accessible, and the datasets accession numbers are listed in the supplementary data. The reference genome sequence H37Rv was retrieved from Genbank database.

### Pre-Processing::

NGS data may encompass sequence artefacts which include poor quality reads, read errors, duplication and adapter/primer contamination which will have an impact on downstream analysis. Therefore, the quality of the data is crucial in distinguishing the true mutations from the sequencing errors otherwise they may lead to wrong conclusions. Pre-Processing of the data was executed using FastQC tool kit to assess the read quality 6.

### Alignment/Mapping:

 For mapping of raw reads versus Mycobacterium tuberculosis h37rv complete genome, BWA-MEM algorithm was used 7. The Flagstats program in Samtools 8 was used to generate statistics on the mapped reads percentage and the duplicates were removed using Mark duplicates in Picard tool.

### Variant Calling and Annotation:

The variants were identified using Genome Analysis Toolkit variant calling best practice workflow including indel realignment and base recalibration 9. This generates output in VCF format, which contains information on the reference allele, alternate allele, and genomic position of variation and quality metrics. Functional annotation of the variants is important to find the link between the disease and genetic variation. SNPeff is an efficient tool to predict the effects of variants, gene annotation, codon change and its impact 10.

### Classification of Isolates:

**Figure 1:**
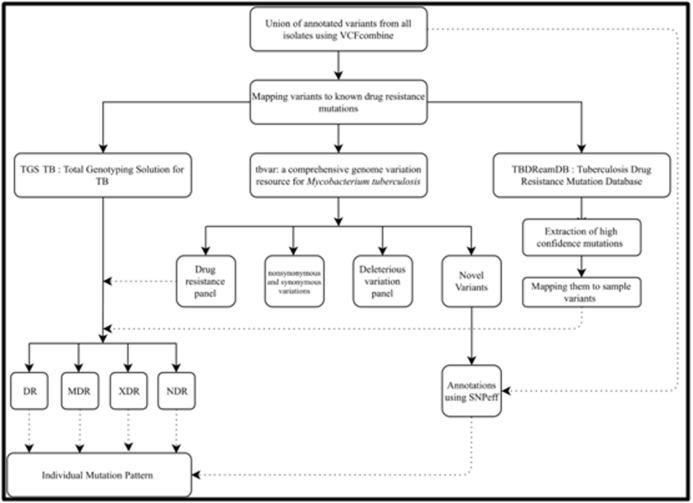
Schematic representation of the pipeline for the classification of drug resistance types

**Figure 2:**
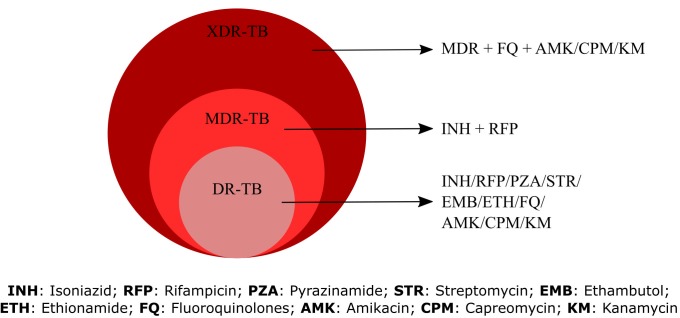
Criteria for classification of the drug resistance types

The annotated VCF files generated from SNPeff were combined using VCFcombine tool from Galaxy web-based platform, which combines all the VCF, files positionally when sites and alleles are identical 11. The variants were then mapped to AMR catalogue used in TGS-TB web-based tool created by TB profiler 12. Further AMR prediction was performed using tbvar: a comprehensive genome variation resource for Mycobacterium tuberculosis and TBDReamDB:TB Drug Resistance Mutation Database 13, 14. tbvar inputs genomic position of variation, allele change information and provides various sections of annotations. These sections include drug resistance panel, which lists the variations annotated to be drug resistance along with the antibiotic and corresponding resistant gene information. Non synonymous and Synonymous variations are listed in a separate panel with predicted SIFT score and the frequency of occurrence of the variation within the population of the samples used to build the database. The sample variations were also mapped to the set of high confidence mutations spanning 49 genes and 9 drugs: Aminoglycosides (Kanamycin/ Capreomycin/ Amikacin/ Viomycin), Ethambutol, Ethionamide, Fluoroquinolones, Isoniazid, Rifampicin, Streptomycin, Pyrazinamide and Para-Amino salisylic Acid downloaded from TBDReamDB. Schematic representation of the pipeline for the classification of drug resistance types is represented in Figure 1. Based on identified drug resistance marker gene variations and corresponding resistance in antibiotics, the samples were classified into DR, MDR, XDR and non-resistant strains based on the criteria mentioned in Figure 2.

## Results and Discussion

### Variant Calling and Classification:

 Out of screening 480 entries in the SRA database search for mycobacterium whole genome data, 74 isolates were selected based on quality control and genome coverage. These samples were further processed for variant calling and the generated VCF files were combined to obtain the union list of mutations positionally. This resulted in identification of 11,130 variants of which 8554 (76.85%) were SNPs, 776 (6.97%) were insertions and 1024 (9.2%) were deletions. These mutations were mapped to the known drug resistant mutations obtained from various databases including TGS-TB, tbvar and TBDReamDB to generate the resistance profiling of each isolate. Further annotation was performed using SNPeffto predict the effects of variants, gene annotation, codon change and its impact for all the variants called. The number of SNPs annotated from various databases arelisted in the Table 1. 3609 novel variations from tbvar database were annotated using SNPeff to obtain the gene annotation and codon variations. The combined analysis of resistance conferring mutations from various databases revealed that among the 74 isolates, 16 were classified as XDR; 16 as MDR; 20 isolates as DR; 6 isolates were found to be non-resistant strains based on AMR predictions and 16 samples had low depth.

### Antimicrobial Resistance Pattern of the Classified ResistantTypes:

**Figure 3:**
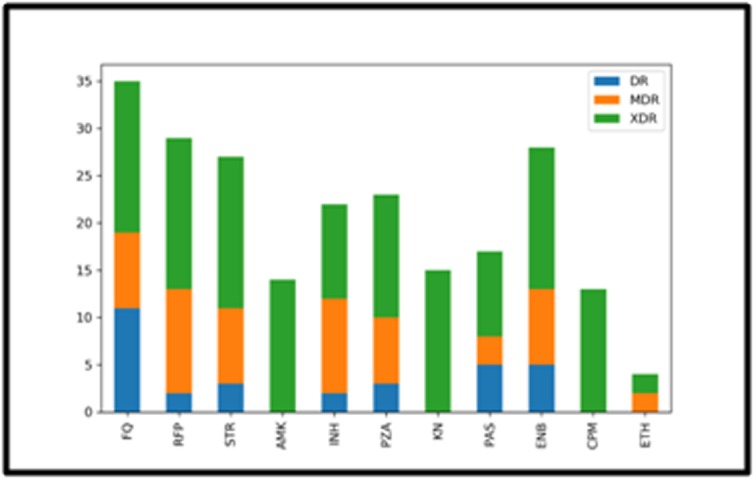
Antimicrobial resistance profiling of classified resistant types

**Figure 4:**
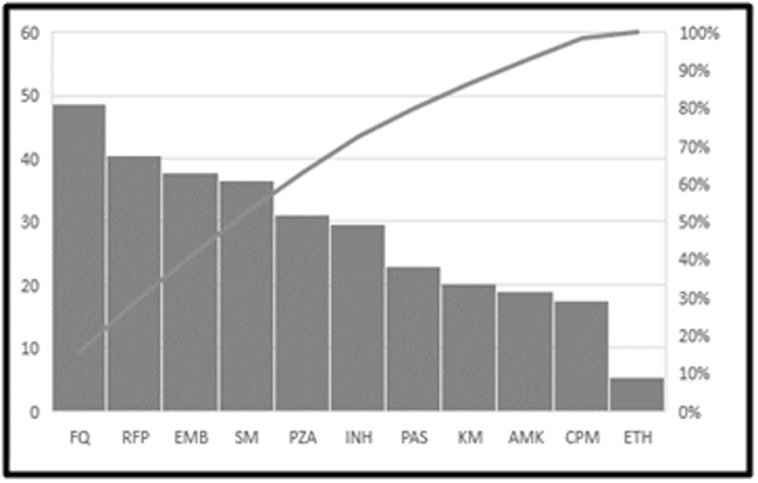
Percentage of isolates resistance to various anti-TB drugs

Antimicrobial resistance pattern was determined based on mutations conferring drug resistance to anti-TB drugs. The determined resistance pattern for XDR, MDR and DR strains are illustrated in Figure 3. XDR classified isolates showed resistance to all the compared drugs in the study supporting the classification. The percentage of drug resistance for the individual drug was determined and is represented in Figure 4. First-line antituberculosis drugs are catalogued as Group 1 consisting of resistance to Isoniazid, rifampicin, ethambutol, and pyrazinamide. In our dataset, 60 % of the isolates were resistant to group 1 antituberculosis drugs. Second-line anti-tuberculosis drugs were analysed and found that 47.29% of isolates were resistant to Group 2 consisting of fluoroquinolones; 21.62 % isolates resistant to Group 4 consisting of Amikacin, capreomycin, kanamycin; 5.4% isolates resistant Group 5 consisting of ethionamide and 22.97% isolates resistant to Group 6 consisting of para-amino salicylic acid drug resistance 15. Profiling of drug resistance and susceptibility will help to decide the drug regimen and dosage levels.

### Genomic Mutation Pattern in Different Resistant Types::

**Figure 5:**
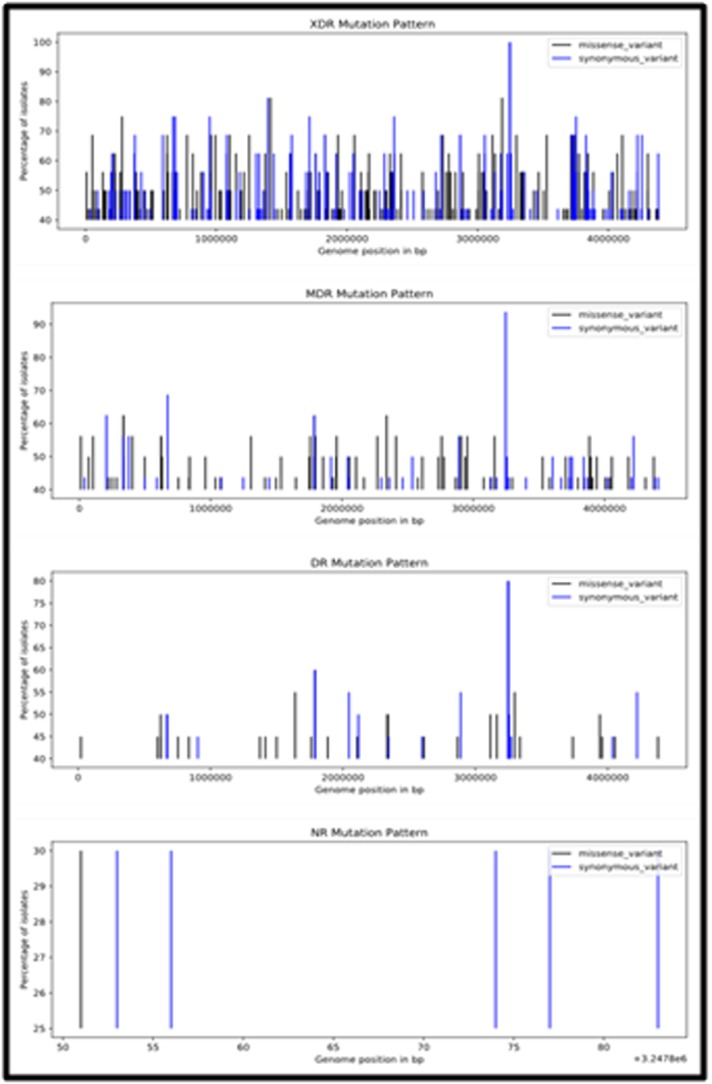
Mutation pattern in classified resistant types

**Figure 6:**
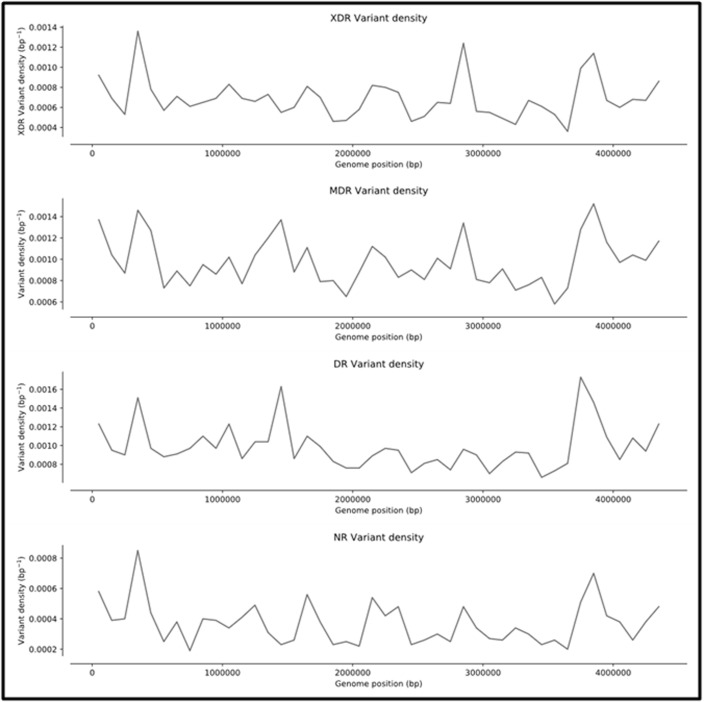
Variant density plot of DR, MDR, XDR and NR isolates across the genome

The annotated SNPs of the predicted resistant types were combined to obtain the list of mutations specific to each resistant type. Python script was written to read the annotated VCF files and to count the frequency of synonymous and missense mutation across the genome to derive the mutation pattern which is represented in Figure 5. This graph explains the distribution of SNPs for individual drug resistant type across the genome. The mutation pattern will provide a graphical visualization of variations and conserved regions in the genome to be compared between the strains.The pattern was differentiable between the classified strains, showing high number of mutations in XDR classified strains and less denser variations in non-resistant type.The SNP density across the genome with a window size of 1,00,000 bp showed the least variations density values in the non-resistant types and higher values in the resistant strains (Figure 6).

### Hotspot Mutations in Candidate Genes:

**Figure 7:**
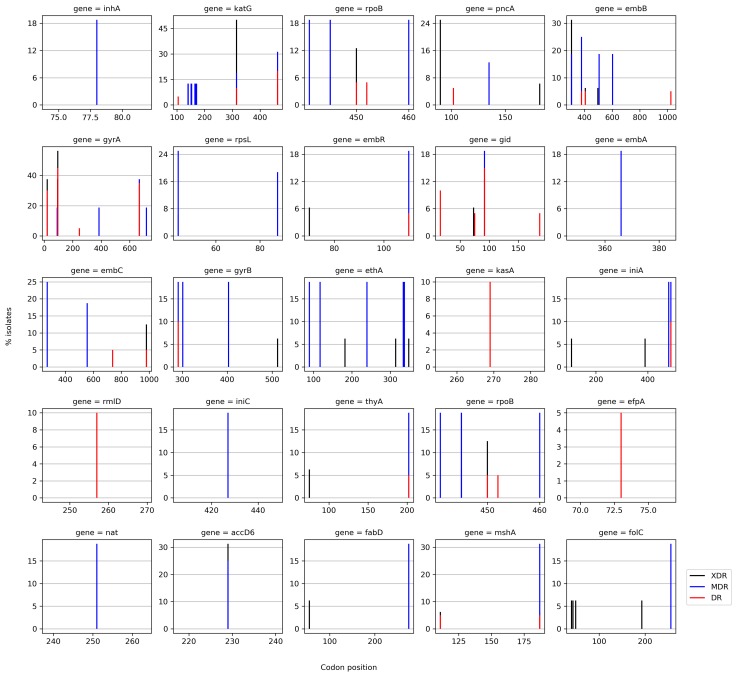
Identified hotspot mutations in candidate genes

Identification of amino acid changes is crucial to understand the association of resistance with drugs. Python script was written to generate the pattern of codon variations in 25 candidate drug resistant genes 4, considering only missense mutations. Each codon variation in the respective candidate gene explains evolution of resistance to specific drug. The percentage of isolates carrying known codon variations in the hot spot regions in XDR, MDR and DR isolates are depicted in Figure 7. The identified codon variations were compared with the previously reported variations and are tabulated in the Table 2. The novel variations around the hot spot regions with unknown drug resistance mechanism are also plotted in Figure 7. This evidence of association between codon variation and the resistant strains can be used further in targeted mutation screening for identification of drug resistance and non-drug resistant regions can be new targets for drug discovery process. The demonstrations of codon variation in hotspot region and also outside resistant determining region will have implications in diagnostics of TB and drug development process 23.

## Conclusion

The present study explains the classification of drug resistant strains based on the known drug resistance mutations obtained from various TB mutation databases. The mutation pattern generated for the classified strains helps to understand the distribution of the SNPs in certain genomic regions resulting from the drug selection pressures, thus providing the information on evolutionary targets of drug resistance mechanism. Profiling resistance to various TB drugs is important to decide the drug regimen. Otherwise, a faulty diagnosis leading to the ineffective regimen will further increase the development of Antimicrobial Resistance. The schematic representation of codon variations gives overall picture of resistance regions in the candidate genes. The hot spot regions will serve as diagnostic tool for screening resistance and non-drug resistant regions can be alternative drug targets to combat resistance. Rapid and accurate prediction of drug resistance through molecular diagnostics promise to improve patient's treatment outcome. In future directions, implementation of molecular based diagnosis in the clinical setting will help in timely diagnosis and efficient treatment of TB patients will reduce the development of AMR.

## Conflict of Interest

Authors declare no conflict of interest.

## Figures and Tables

**Table 1:** Number of SNPs annotated from various databases

Variant Type	tbvar	SNPeff	Novel_tbvar_Snpeff_annotation	AMR catalogue (TGS-TB)
Novel Variants	3609			
Drug Resistance variants	18			45
Synonymous Variants	1613	3027	1439	
Non-Synonymous Variants	2645	4141	1544	
Deleterious Variations	618			
Stop Lost	17	5	2	
Stop Gained	37	79	33	

**Table 2:** Identified codon variations in comparison with previously reported studies

Resistant Type	Gene	Codon variation	Frequency of mutation	Reference
XDR	gyrA	S95T	56.25%	16
		E21Q	37.50%	
		G668D	37.50%	
		D94G	37.50%	
	katG	S315T	50%	17
	accD6	D229G	31.25%	18
	embB	M306I	31.25%	19
MDR	katG	R463L	31.25%	20
	mshA	A187V	31.25%	
	embB	E378A	25%	21
	embC	T270I	25%	
	accD6	D229G	25%	22
	rpsL	K43R	25%	
DR	gyrA	S95T	45%	16
		G668D	35%	
		E21Q	30%
